# Imaging for the diagnosis of acute myocarditis: can artificial intelligence improve diagnostic performance?

**DOI:** 10.3389/fcvm.2024.1408574

**Published:** 2024-08-29

**Authors:** Vijay Shyam-Sundar, Daniel Harding, Abbas Khan, Musa Abdulkareem, Greg Slabaugh, Saidi A. Mohiddin, Steffen E. Petersen, Nay Aung

**Affiliations:** ^1^ William Harvey Research Institute, Queen Mary University of London, London, United Kingdom; ^2^ Barts Heart Centre, St Bartholomew’s Hospital, London, United Kingdom; ^3^ Digital Environment Research Institute, Queen Mary University of London, London, United Kingdom; ^4^ School of Electronic Engineering and Computer Science, Queen Mary University of London, London, United Kingdom

**Keywords:** machine learning, artificial intelligence, cardiac MRI, myocarditis, diagnosis

## Abstract

Myocarditis is a cardiovascular disease characterised by inflammation of the heart muscle which can lead to heart failure. There is heterogeneity in the mode of presentation, underlying aetiologies, and clinical outcome with impact on a wide range of age groups which lead to diagnostic challenges. Cardiovascular magnetic resonance (CMR) is the preferred imaging modality in the diagnostic work-up of those with acute myocarditis. There is a need for systematic analytical approaches to improve diagnosis. Artificial intelligence (AI) and machine learning (ML) are increasingly used in CMR and has been shown to match human diagnostic performance in multiple disease categories. In this review article, we will describe the role of CMR in the diagnosis of acute myocarditis followed by a literature review on the applications of AI and ML to diagnose acute myocarditis. Only a few papers were identified with limitations in cases and control size and a lack of detail regarding cohort characteristics in addition to the absence of relevant cardiovascular disease controls. Furthermore, often CMR datasets did not include contemporary tissue characterisation parameters such as T1 and T2 mapping techniques, which are central to the diagnosis of acute myocarditis. Future work may include the use of explainability tools to enhance our confidence and understanding of the machine learning models with large, better characterised cohorts and clinical context improving the diagnosis of acute myocarditis.

## 1 Introduction

Myocarditis is characterised by inflammatory cell infiltration into the myocardium associated with a high risk of heart failure (1). Heterogenous aetiologies include direct injury following cardiotropic viral infections, non-viral infective causes, drug reactions, as well as organ-specific or multi-system autoimmunity. Regardless, immune mediated damage, often mediated by infiltrating lymphocytes, is central to myocardial injury (2). The annual occurrence of myocarditis has been estimated to be 1.8 million cases with the prevalence ranging from 10 to 106 per 100,000 worldwide (3). There were 131,376 years lived with disability and 1.26 million years of life lost attributable to myocarditis in 2017 in the Global Burden of Disease Study (3). Increasingly, myocarditis is diagnosed more frequently with impact on a wide range of age groups including young people (4). There are diverse modes of presentation including via pathways for acute coronary syndromes, life-threatening cardiac arrhythmias/sudden death, and acute/chronic heart failure. Clinical outcomes include complete recovery, the consequences of chronic stable myocardial injury, or those attributable to chronic relapsing inflammatory states. This clinical variability poses significant diagnostic challenges including: establishing when to make a diagnosis in the patient pathway, ascertaining key diagnostic features and evaluating their respective clinical utilities, and achieving the aim(s) of the diagnostic algorithm (e.g., to distinguish myocarditis from myocardial infarction, or between chronic myocarditis and other causes of a dilated cardiomyopathy).

Cardiac magnetic resonance (CMR) imaging is considered by many to be the preferred non-invasive investigation for the diagnosis of myocarditis. It is often under-utilised due to complex protocols and contrast requirements as well as the lack of reporting expertise and high expense (5, 6). CMR can detect hyperaemia, myocardial oedema, and interstitial expansion; however, these changes are not specific to myocarditis and will accompany any cause of acute myocardial injury, emphasising the importance of a well-defined clinical context when interpreting imaging results. In contrast to endomyocardial biopsy (EMB), CMR cannot detect causes of the inflammatory disorder, and cannot characterise the inflammatory infiltrate mediating myocardial injury.

Although considered to be the reference standard for diagnosis, EMB is performed in only a minority of suspected cases (5, 7)_._ This is in part due to its invasive nature and fear of complications such as ventricular rupture, and a low diagnostic yield due to sampling error and false negatives (8).

CMR has been validated against EMB-derived histology with significant correlations between fibrosis and scar on non-invasive assessment and histological fibrosis (9). Current consensus opinion is to use CMR in all suspected cases with additional multi-modal imaging including ^18^F-fluorodeoxyglucose positron emission tomography (FDG-PET) in certain clinical contexts such as sarcoidosis (10, 11). Despite the existence of current imaging-based consensus guidelines such as the Lake Louise Criteria (LLC), they are not consistently applied in clinical practice. Due to issues around sampling inaccuracies underlying tissue characterisation sequences and incomplete coverage of myocardium during CMR acquisition, clinical CMR reporting of myocarditis often does not conform to the LLC (5). Despite decades of research into myocarditis, the prognosis is poor in the group of patients presenting with heart failure or arrhythmias, highlighting the need for systematic analytical approaches to enhance diagnosis and risk stratification (1).

Machine learning (ML)—a subset of artificial intelligence (AI)—is a collection of techniques that enable computers to learn tasks from data. ML and AI applications are increasingly prevalent in CMR (12, 13). For example, ML is reported to match diagnostic human performance and augment phenotyping, with the added benefit of discrimination between acute and chronic myocarditis (14, 15). In addition, AI analytical tools may be more robust for decision making, augmenting clinical workflows, saving clinicians time and aiding diagnosis (16).

In this article, we will describe the role of CMR and other imaging modalities used for the diagnosis of myocarditis followed by a literature review on the applications of AI and ML in CMR to diagnose acute myocarditis.

## 2 The role of imaging in the diagnosis of myocarditis

### 2.1 Current role of cardiovascular magnetic resonance in myocarditis

Multi-parametric measures provided by CMR are typically used to diagnose myocarditis. Those outlined by the LLC working group were most recently updated in 2018 to reflect evidence and advances in CMR technology including T1 and T2 mapping. The main diagnostic criteria are the presence of at least one CMR T1-based marker for non-ischaemic myocardial injury [T1 mapping, extracellular volume (ECV), or late gadolinium enhancement (LGE)] and one T2-based marker for myocardial oedema (T2 mapping or T2-weighted imaging). The supporting criteria include left ventricular systolic dysfunction and/or pericarditis or pericardial effusion. For T2-weighted sequences, either signal hyperintensity in T2-STIR sequences or if no focal signal change, the use of global myocardial T2 to skeletal muscle T2 signal intensity ratio ≥2 fulfils one major criterion for acute myocarditis (17). Mid-wall LGE and T2 elevation correlate with worse outcomes (18–20). The assessment of LGE pattern and oedema specific sequences such as T2-weighting and T2 mapping are currently used to support the diagnosis of acute myocarditis in clinical practice. One study reported that a normal ECV appears to be the most sensitive imaging biomarker in the acute setting of myocarditis to exclude this as a diagnosis (21), although further evidence on its additional value over T1/T2 mapping techniques is needed. Myocardial strain using feature tracking adds incremental prognostic value beyond clinical features, LGE and the left ventricular ejection fraction (22).

Additionally, CMR-based radiomics and texture analysis are emerging as promising imaging markers to aid the diagnostic work-up of myocarditis (14). Prior research has shown that tissue tracking is a significant predictor of LGE in myocarditis when there is preserved LV function and no visually-detected regional wall motion abnormalities (23). This study did not employ machine learning methodology, instead using receiver operating curves and net reclassification analyses to assess the predictive yield of 2D and 3D tissue tracking derived strain parameters. Whilst there are issues with reproducibility, using tissue tracking parameters alongside other measures may be useful in those patients with a contra-indication to contrast-enhanced CMR.

Global longitudinal strain (GLS) on CMR is sensitive to changes in cardiac function and has been shown to be an independent prognostic indicator associated with adverse outcomes and may improve risk stratification of those with acute myocarditis (22). GLS also shows significant correlations with CMR parameters of myocardial oedema (24).

There is no consensus in how CMR should be used in the monitoring of myocarditis including the role for serial CMR scans and data on the best time-interval to perform follow-up scans to evaluate therapy response. CMR cannot directly detect inflammation but instead can detect changes in T1 and T2 which are surrogate markers for fibrosis and oedema. Fibrosis is more commonly observed in chronic rather than acute myocarditis. Experimental models of myocarditis show that findings in the acute phase parallel that seen in humans and fibrosis occurs in chronic rather than acute myocarditis (25, 26).

T1 and T2 mapping are promising tools for discriminating between the acute phase and the healed phase of myocarditis or diagnosing those with chronic symptoms given their higher sensitivity for detection of myocardial oedema and fibrosis (27, 28). T2 mapping appears to be more effective than T1 mapping at detecting residual inflammation at four to eight weeks of follow up (27). The MyoRacer Trial (28) also demonstrated similar results but is complicated by shortcomings in methodology including sampling errors from EMB and T1 values that are not comparable to those reported elsewhere with similar CMR acquisition protocols. Further research is required to assess whether T1 mapping may be suitable for diagnosing those with chronic myocarditis and T1 mapping as a imaging biomarker for diffuse myocardial fibrosis offers a ML target (29). Using CMR to detect fibrosis may reflect cases which have progressed from the acute to the chronic phase and those with acute myocarditis with complete resolution may be missed by CMR.

An objective CMR based inflammation score may aid standardisation of CMR reports and could potentially be calculated using quantitative assessment of the CMR surrogates reflecting myocardial strain, diffuse myocardial fibrosis and oedema (30). ML algorithms may be well suited to analyse all available data and allow the generation of an imaging-based inflammation score, promoting novel diagnostic classifications of myocarditis. Such scores may also have utility in following disease progression, monitoring for features indicating recovery as well as those providing early indicators of relapse.

In high-risk cases such as fulminant myocarditis with cardiogenic shock, patients may be clinically unstable or unsuitable for CMR with some requiring haemodynamic support (11). In this scenario, a standard CMR protocol with contrast is not feasible due to the risk of deterioration with arrhythmia and heart failure sequelae. Application of ML on non-contrast data to predict LGE may permit a shorter total CMR acquisition time helping to guide decisions regarding diagnostics (including EMB or FDG-PET) and acute therapy (e.g., immunosuppression, extracorporeal oxygenation and mechanical support, transplantation).

### 2.2 The complementary role of ^18^f-fluorodeoxyglucose positron emission tomography to cardiovascular magnetic resonance in myocarditis

FDG-PET is an alternative to CMR for detecting focal and diffuse myocardial inflammation (11). It directly measures the increased glucose metabolism which results from the activation of the local inflammatory infiltrate. A simultaneous hybrid PET/CMR approach may yield an accurate diagnosis in cardiac sarcoidosis and should be considered in the assessment of the presence of disease and stage of disease (31).

There is a paucity of high-quality evidence including large clinical trials to support the routine use of FDG-PET in the acute setting (32, 33). One study reported the use of FDG-PET in the diagnosis of myocarditis, and to guide withdrawal of immunosuppressive therapy (34). In this study, 75 patients with acute myocarditis and ventricular arrhythmia were enrolled prospectively, and all underwent EMB and CMR evaluated according to the LLC. FDG-PET was obtained either when there was a contra-indication to CMR, or if there were discordant EMB and CMR findings. Lymphocytic myocarditis was diagnosed in 88%, and 55% underwent ICD implantation. FDG-PET and CMR were concordant in classifying 90% of patients with anteroseptal vs. inferolateral patterns of inflammation with 10% discordance in the localisation of these patterns by the different diagnostic imaging modalities. The best match between CMR and those who had a positive FDG-PET test were with standard LLC and T2 short tau inversion recovery (STIR) sequences (R^2^ = 0.739).

This single centre study addressed highly selected myocarditis patients admitted to hospital with ventricular arrhythmias; the findings may not apply to less severe acute disease, or to more chronic/borderline cases.

When compared with FDG-PET as a sole diagnostic imaging modality, there is more widespread availability of CMR and CMR is well suited to AI approaches given multiple facets on image acquisition, reconstruction, segmentation, and quantification which can be improved by ML approaches (35).

### 2.3 Machine learning in CMR relevant to myocarditis

AI in cardiac imaging is an evolving field, with utility extending well-beyond segmentation and quantification. This includes guiding the diagnosis of cardiovascular disease, for example in identification of features of pulmonary arterial hypertension on CMR (36). In the context of myocarditis, the most obvious application of ML to CMR is on scar and oedema quantification. Scar evaluation is an important component of tissue characterisation in CMR, but quantitative assessment can be so time consuming as to limit clinical utility. Automatic segmentation of scar and oedema can be challenging due to the multiple sequences of CMR data acquired at different time points and breath-holds, and difficulties in labelling the data with high interobserver variance making it challenging to derive a consensus ground truth.

Convolutional neural networks (CNNs) are the most frequently used type of deep neural network for image analysis. For example, CNNs have been used to segment the extent of myocardial scar on LGE images (37). A standard CNN typically consists of an input layer, and functional layers which transform the input into a specific form of output. A transformer-based method has been proposed for the classification and segmentation of myocardial fibrosis (38). Transformers are a type of neural network architecture “transforming” input data into output data by learning the relationships between sequences and their context; for example, in myocarditis in the context of high T2 and elevated troponin and thickened myocardial walls, LGE represents oedematous interstitial expansion rather than the mature “replacement” scar in myocardial infarction.

Vision transformers, used in computer vision, can be used for image classification and are a topic of interest (39). There is some debate about the role of CNNs, vision transformers and other architectures such as ConvNext v2 in classification and segmentation (40, 41). Future work may feature hybrid CNN-transformer methods (42). It is important to demonstrate that these ML methods can improve healthcare delivery to encourage their uptake and use in clinical practice. Papetti et al. applied ML methodology to a myocardial infarction cohort, with demonstrable time savings, accuracy and minimal required user input for LV and scar segmentation (37).

## 3 Literature review

**Figure 1:**
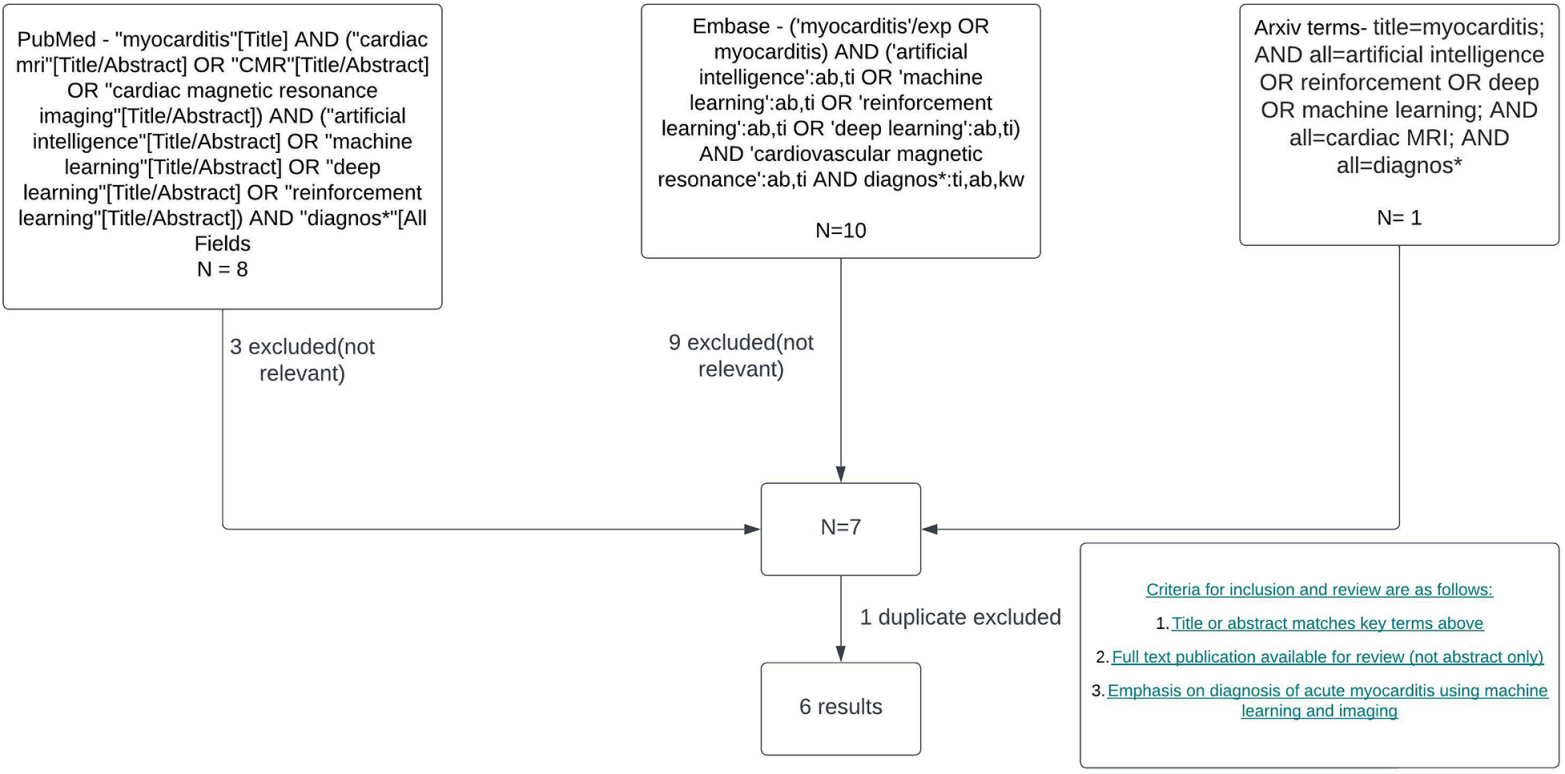
Literature review and filtering flowchart. Created with Lucid.com.

**Figure 2:**
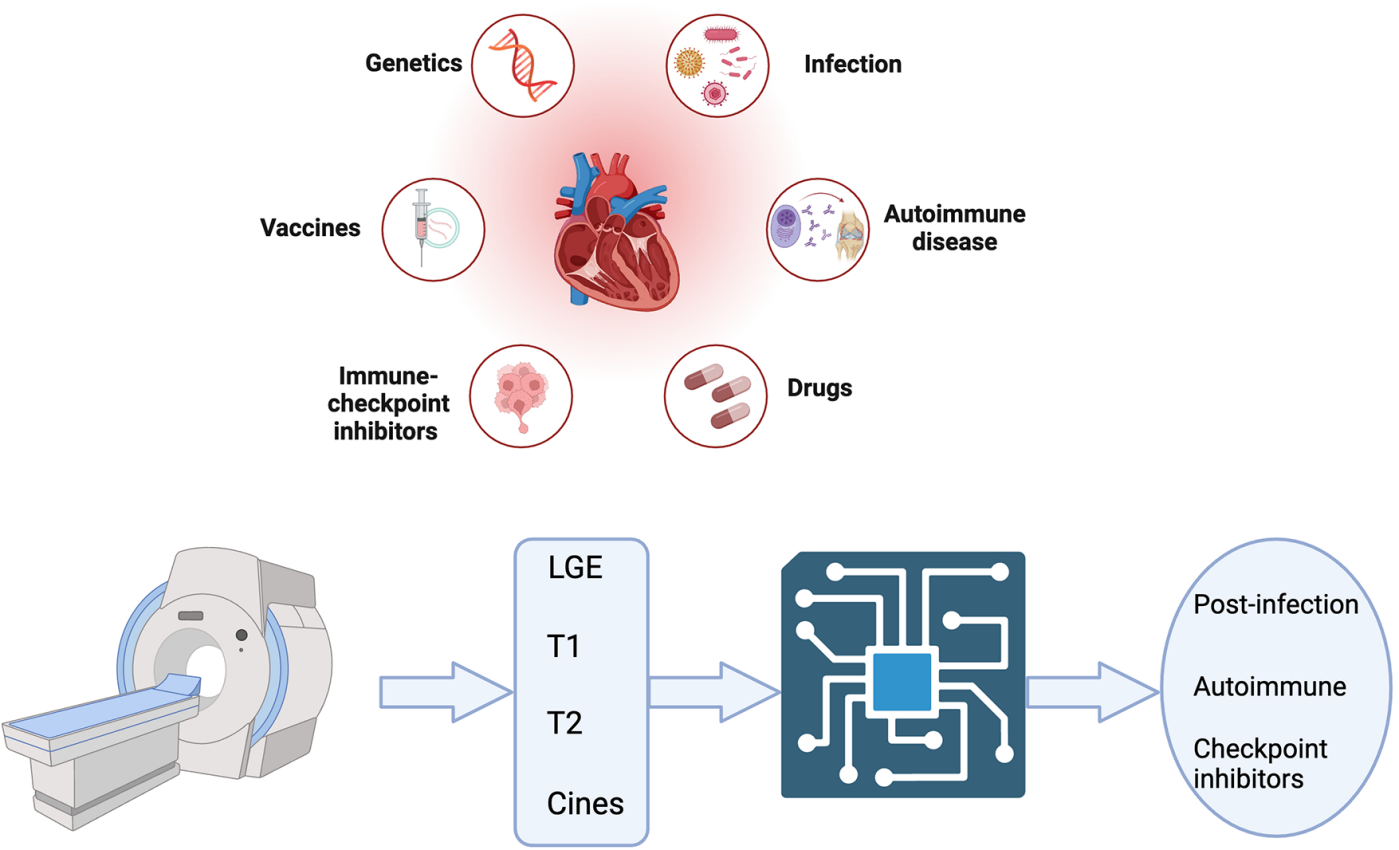
Central illustration created with bioRender.com. A variety of triggers predispose to myocarditis which can be acute and/or chronic. Cardiac MRI features are shown as inputs for machine learning aided diagnosis of acute myocarditis.

The search strategy to identify studies using ML in CMR to diagnose acute myocarditis as summarised in Figure 1 yielded 6 results. Duplicate publications were removed, and manual review of articles was performed against the criteria specified in Figure 1. Various databases including PubMed, Embase, and Arxiv were interrogated. Key terms included myocarditis, CMR or cardiac MRI, machine learning, artificial intelligence, reinforcement learning and diagnosis. Figure 2 summarises the range of causes of myocarditis and the role of CMR and ML in diagnosis.

Table 1 summarises the main objectives, input data, methods and evaluation measures. Three out of the six publications were based on a well-annotated CMR dataset from Tehran. The Tehran dataset is publicly available along with the code for the study by Sharifrazi et al.; however, metadata are not available and the dataset can be considered of uncertain provenance (45, 49). Patients with a clinical suspicion of myocarditis only underwent CMR if it was thought likely that this test could determine patient management which may have resulted in selection bias. Healthy controls were included in the final dataset, and the models are developed and tested on their ability to differentiate between disease and normal controls. In reviewing these studies, it must not be forgotten that the “real-world” objective of diagnostic algorithms includes detecting inflammation and distinguishing between different causes of myocardial injury—and not simply discriminating between a specified disease and normal controls.

**Table 1:** Summary of identified publications for this review.

Objective	Publication date	Authors	Input data (CMR sequences)	Dataset	Methodological considerations	Sample	Evaluation
To distinguish between myocarditis and MI	19.12.19	Di Noto et al. (43)	Cines, SSFP, T2, LGE	University Heart Center, University Hospital Zurich	Exponential filter, wavelet transform filter, recursive feature elimination	173 patients (111 MI, 62 Myocarditis)	Training (90%); Testing (10%). Nested 10-fold cross validation
To predict the presence of LGE in myocarditis patients	16.09.22	Cavallo et al. (44)	Short Axis (STIR, PSIR), T1, T2, LGE	Policlinico Tor Vergata, Rome, Italy	Weka data mining platform Correlation-based Feature Selection Ensemble ML	19 patients	Training (70%); Testing (30%) 10-fold cross validation
To distinguish myocarditis from healthy controls	04.01.22	Sharifrazi et al. (45)	Cine, T1, T2, LGE (PSIR)	Z-Alizadeh Sani myocarditis dataset	Convolutional neural network combined with k means clustering	32 myocarditis patients, 15 healthy controls	Training (90%); Testing (10%)
To distinguish myocarditis from healthy controls	30.06.22	Moravvej et al. (46)	Cine, T1, T2, LGE (PSIR)	Z-Alizadeh Sani myocarditis dataset	Reinforcement learning with population-based weights strategy	586 myocarditis patients, 307 healthy controls	5-fold cross validation
To distinguish myocarditis from healthy controls	26.10.22	Jafari et al. (47)	Cine, T1, T2, LGE (PSIR)	Z-Alizadeh Sani myocarditis dataset	Turbulence Neural Transformer (TNT) architecture Explainable-based Grad Cam method	32 myocarditis patients, 15 healthy controls	10-fold cross validation
To distinguish myocarditis from healthy controls	08.04.22	Ghareeb et al. (48)	Cines, T1, T2, LGE(PSIR)	Heart Hospital, Hamad Medical Corporation, Qatar	Unsupervised learning- K means clustering, Bayesian factor analysis	169 patients	Silhouette score

NB The reported sample sizes differ between the 3 studies using the Z-Alizadeh Sani myocarditis dataset– we are unable to ascertain the reasons for this and it is unclear in the manuscripts.

### 3.1 Clustering with supervised learning

The study by Sharifrazi et al. classified images in the Tehran dataset using a hybrid CNN and k-means clustering algorithm (CNN-KCL) (45). After the dataset was pre-processed, images were placed in several clusters with similar images placed in a cluster and each cluster utilised by the CNN for classification of myocarditis. No clear justification is given for why clustering is necessary—the data is fully labelled which would suggest a supervised learning approach would suffice. Initially, two clusters of healthy and myocarditis were given as inputs to the model. Due to heterogeneity in the input data the initial accuracy of the model was low. The data was divided into more clusters (a total of four clusters with myocarditis and healthy each divided into two clusters) with similar images categorised in the same cluster representing more distinct patterns. The CNN-KCL approach significantly outperformed other traditional ML approaches such as decision trees and random forests for this classification task. Model performance metrics are summarised in Table 2. The accuracy of this model was 97.41%, but the clusters used in the model are of uncertain clinical significance and the diagnostic aim of this model differs from that used in clinical practice as described previously.

**Table 2:** Model performance metrics (%) for the diagnosis of acute myocarditis in CMR.

Models	Accuracy	Precision	Recall	Specificity	F1-score	AUC
CNN-KCL [Sharifrazi et al. (45)]	97.41	97.6	95.7	98.56	96.5	97.05
RLMD-PA [Moravejj et al. (46)]	88.6	84	86.3	90.1	85.1	N/A
TNT [Jafari et al. (47)]	99.68	99.47	99.59	99.72	99.53	99.94
Inception v4 [Jafari et al. (47)]	99.7	99.44	99.69	99.71	99.57	99.94

NB for RLMD-PA, G means (88.2%) instead of AUC was calculated.

CNN-KCL, convolutional neural network k-means clustering; RLMD-PA, reinforcement learning-based myocarditis population-based algorithm; TNT, turbulence neural transformer.

### 3.2 Reinforcement learning

In the study by Moravej et al., the authors attempted to address concerns with CNN-KCL using the artificial bee colony (ABC), a population-based algorithm and the reinforcement learning (RL) method with the Tehran dataset (46). One concern relates to the pre-processing stage, where the image matrix was considered as a vector in k-means, resulting in missed pixels around a specific pixel. This may have led to the loss of relevant input imaging data to the model. RL allows the model to learn through trial and error using feedback from its actions. The main advantage of this RL approach is that it's fully supervised as opposed to the CNN-KCL approach which combines both supervised and unsupervised methods and may not perform as well. ABC emulates the foraging behaviour of bees to arrive at an optimal result. Classification of images was thought of as a sequential decision-making process with the initial optimal weighting values calculated using reinforcement learning-based myocarditis diagnosis combined with a population-based algorithm (RLMD-PA) to address the problem of imbalanced data.

Table 2 shows the model performance metrics for RLMD-PA. This outperformed conventional and metaheuristic algorithms with improvements in the error in recall (25%) and F-score (22%). The RLMD-PA model reduced error by more than 43% with mean performance metrics superior to other methods such as CNN-KCL, CNN + RL, and CNN + ABC. This study presents a new model for classifying myocarditis images and showed that RLMD-PA was an effective classifier for myocarditis images.

### 3.3 Deep learning and explainable artificial intelligence

Jafari et al. applied a deep learning (DL) model to the Tehran dataset to diagnose myocarditis (47). Pre-processing steps included filtering, resizing and use of the data augmentation methods, CutMix and MixUp to create new training examples and synthetic CMR images (50, 51). CutMix uses patches from one image and pastes them onto another image, whilst MixUp uses linear interpolation to combine two images. These strategies can reducing the risk of overfitting by creating variations and improving the diversity of the training set that the model can learn from and improve generalisability to unseen data (52). Pre-trained models and transformers were then used for feature extraction and classification of the synthetic CMR data. Pre-trained models are CNN architectures based on supervised learning that have been trained on large datasets, including the ImageNet dataset. This can then be applied to myocarditis and other cardiovascular disease datasets which often have small numbers of subjects through transfer learning approaches (53). Jafari et al. aim to build image classification models with several architectures analysed in this study (47).

Various pre-trained models for diagnosis using CMR images were compared with and without data augmentation. Inception V4 and Turbulence Neural Transformer (TNT) were most successful, and the metrics of these models are shown in Table 2.

One of the main criticisms of ML and DL is their “black box” nature. The authors attempted to address this by using explainability techniques, a form of interpretability methods. These can be used to extract relevant knowledge from the image data illustrating how a given model yields individual predictions. Jafari et al. used explainable AI as a final step in post-processing (47). The grad-cam method was used and helped to visually identify suspicious areas on imaging that could potentially help clinicians to diagnose myocarditis in the early stages (54).

All three studies that use the Tehran dataset have limitations, and these include those that derive from the dataset's relatively small size, its single centre nature, and the lack of external validation. Furthermore, there was evidence of selection bias and an absence of other cardiovascular disease controls. Patient-level data, including baseline characteristics, are not available. Variance in demographic factors including medical conditions, sex and age may also influence the results and their generalisability.

### 3.4 Supervised and unsupervised learning with radiomics

A study by Di Noto et al. compared the diagnosis of myocardial infarction (MI) vs. myocarditis using CMR-based radiomic features from LGE (43). The diagnosis of MI was based on clinical presentation, ECG and laboratory findings, whilst the diagnosis of myocarditis was also defined by clinical parameters including two out of the three LLC (5). CMR sequences included steady state free precession cines, T2-weighted images in three short-axis slices (using a double-inversion black-blood spin-echo sequence) and LGE images in three short-axis slices. LGE images were analysed by two independent readers for discrimination between myocarditis and MI. Both supervised [recursive feature elimination (RFE)] and unsupervised [prinicipal component analysis (PCA)] feature selection techniques were applied to the radiomics of LGE on retrospective data. Feature selection can simplify the model, reduce training time, and improve model generalisability. Five different ML algorithms from different classifier families were trained: k-nearest neighbour, linear discriminant analysis, multilayer perceptron, support vector machine and TreeBagger. As expected, better performance metrics were obtained for the more experienced reader compared to a less experienced reader for subjective visual analysis discriminating myocarditis and MI.

Performance of the feature selectors was defined by classification model metrics including accuracy, sensitivity, specificity and precision but only reported for RFE feature selection of 2D features and PCA guided selection of 3D features which led to the highest performances. The more experienced reader ultimately performed better than ML in distinguishing MI from myocarditis, whilst the less experienced reader was outperformed by the ML algorithm.

### 3.5 Predicting late gadolinium enhancement using radiomics and supervised machine learning

A study by Cavallo et al. was the first to use radiomics in STIR sequences to predict the presence of LGE in myocarditis, and also aimed to develop abbreviated scan protocols for patients with suspected acute myocarditis (44). Prediction of LGE from non-contrast data has been studied for MI (55). The investigators studied 19 patients that underwent CMR for clinically suspected myocarditis within 30 days of symptom onset. 228 STIR and PSIR images were analysed with 57 images excluded due to suboptimal quality or non-diagnostic images. The mean time from the start of the examination to the last slice of the PSIR sequence was 46.13 ± 11.16 min.

The authors performed an additional analysis on 19 age- and sex-matched patients with normal STIR and LGE imaging, in order to demonstrate that noise or artefact was not affecting the data. After training, ML models with accuracy of greater than 70% were selected and then the probability of presence of LGE was calculated on the test set. These models were grouped together using an Ensemble ML model averaging the predictions obtained. Six radiomics features showed statistically significant differences. The Ensemble ML model showed accuracy of 0.75, sensitivity of 0.80 and a specificity of 0.73 with a NPV of 0.81 and a PPV of 0.70 and AUC of 0.79 (95% CI: 0.66–0.92). The study outlined the population characteristics including age, sex, biochemical data and past medical history. Age and sex were comparable between controls and myocarditis patients. Limitations of this study include the retrospective nature of the study, a small sample number and data from a single MRI scanner. There is also the possibility that the sample may have included patients with undetected coronary artery disease, although none of the LGE images contained LGE in a subendocardial pattern.

### 3.6 Unsupervised machine learning to assess cardiac magnetic resonance inflammation patterns

A study by Ghareeb et al. used unsupervised ML and Bayesian factor analysis to investigate patterns of inflammation (48). The investigators included an ethnically diverse cohort that was retrospectively identified with acute myocarditis detected by CMR and defined by LLC. The exposures studied were LGE and T2 mapping as surrogates for inflammation and oedema respectively. The primary outcome and aim of this study was a clinical combined endpoint of cardiac death, arrhythmia, and dilated cardiomyopathy. The relationship between demographics and geographical differences and CMR parameters including LGE, T2, T1 and T2 mapping was investigated in a separate exploratory analysis.

Anteroseptal inflammation was associated with worse outcomes which is consistent with previous research including as the ITAMY study (18). Geographical location of the patient not appear to explain the variance of CMR inflammation patterns. However, sex did appear to influence inflammation pattern with females (22% of the cohort) less likely to have oedema by T2 mapping and sub-epicardial inferolateral inflammation.

K-means clustering indicated that these patients could be divided between two predominant clusters. One had elevated T2 alongside subepicardial LGE in anterior and inferolateral segments. The other predominant cluster were more likely to have early gadolinium enhancement (EGE), elevated T1 and T2 on parametric mapping and mid-wall and infero-lateral LGE. However, there is overlap between the two clusters with poor delineation. This approach requires some degree of clinical interpretation which is informed by the Bayesian factor analysis.

## 4 Discussion

### 4.1 Limitations in current literature

Current literature highlights the relative paucity of ML applications in imaging to aid the diagnosis of acute myocarditis. Limitations include cohort and control size with cohort characteristics often unavailable and lack of relevant controls. Cavallo et al. predicted LGE data utilising radiomics but this was based on cases alone: the same approach may have limited utility if applied to the more common clinical objective of distinguishing acute myocarditis from other cardiovascular diseases such as MI or heart muscle disease (44). In the interesting paper published by Ghareeb et al., the number of cardiac events was low and further inferences are challenging given the size of the cohort (48). Additionally, although comparisons between the clusters was completed with factor analysis these are statistically underpowered. All the reviewed papers analyse imaging data from single centres, and in some studies the sample size is very small with the risk that models may be overfitted to the training data.

As outlined, T1 and T2 are important CMR parameters in the diagnostic imaging evaluation of myocarditis. T1 and T2 were not included in analyses (Di Noto et al.) or not part of the imaging dataset (Ghareeb et al.) (43, 48). In the paper by Di Noto et al., segmentation was performed by an individual as opposed to by semi-automated or automated processes (43). Future CMR studies are likely to incorporate more automation of segmentation of multiple tissue characterisation parameters given the inaccuracies with manual processing of images.

The available publications demonstrate a variety of approaches including supervised, unsupervised and reinforcement learning but there are limitations with the AI/ML methods utilised. Jafari et al. whilst utilising novel techniques in the context of myocarditis diagnosis are applying existing algorithmic architectures and methods developed for use in different cardiovascular conditions rather than a specialised approach to myocarditis. Generalisability to an external dataset and patient groups in other hospitals/regions may be challenging. Given that the Tehran dataset is fully labelled, the combination of supervised and unsupervised techniques used by Sharifrazi et al. seems unnecessary where a fully supervised learning method would suffice (45). The RL approach used by Moravejj et al. is fully supervised but seems arbitrary and overly complex and not comparable to other alternative approaches such as CNNs (46).

In general, confidence in AI/ML tools and their applicability is an ongoing area of debate and it will be important to exploit more recent techniques and compare them with the Grad cam method which was used in the paper by Jafari et al. (47). Explainable Artificial Intelligence (XAI) plays a vital role in enhancing human understanding and trust in DL systems and as models get larger with more widespread uses explainability will minimise the adverse consequences of model mistakes. At present, XAI including that used in healthcare tends to depend on a single explainer with the risk of disagreement in explainability methods systematically applied to the same dataset.

Myocarditis varies by sex and age, with acute myocarditis occurring predominantly in young males while limited data suggests women get myocarditis post menopause (56). An understanding of age and sex differences is critical for improving the efficacy of diagnosing acute myocarditis using ML. Training data will likely be imbalanced in terms of sex distribution which reflects the natural prevalence of myocarditis. Accordingly, the training model may not generalise well to models with female subjects.

Existing AI models are trained on physician interpreted CMR datasets. CMR has limitations in achieving myocarditis diagnosis including the inability to determine aetiology unlike this is a barrier towards routine clinical implementation of AI in myocarditis. The complexity and unfamiliarity of AI coupled with the heterogeneity in myocarditis will compound the above with regards to difficulty in achieving user clinician confidence in “AI for myocarditis” as a tool to advance patient care.

### 4.2 Future work

There are several ways in which the issues above may be mitigated. Large well characterised cohorts incorporating greater clinical context and long term follow up as additional variables alongside CMR image data may lead to better predictions with a more accurate diagnosis of myocarditis. In addition, pooling image data in a federated fashion, with individual centres training their models on larger datasets may lead to more robust results. A well curated publicly available dataset will allow algorithms to benchmark against comparable methods aiming to achieve similar AI/ML imaging objectives. Data augmentation in the pre-processing of CMR images may assist by generating synthetic CMR images which can aid model predictions. Combining CNNs using an ensemble method might lead to higher model performance. It is also the case that AI-derived clustering could allow novel ways of stratifying myocarditis with the potential to conduct outcome studies based upon this approach.

Explainability tools may supplement the intuitive understanding of AI/ML. We envisage judicious application of post-hoc XAI methods such as Grad-cam to address “black box” criticisms of ML models. A more intuitive human-oriented approach might be more helpful to clinicians with arrows and text indicating and justifying the diagnosis. As far as we are aware, there are no clinically validated studies of XAI in clinical cardiology practice and no XAI methods have undergone regulatory approval. Demonstrating that the predictions of ML models can be both explained and the models themselves are interpretable will aid integration into clinical diagnostic workflows in cardiology. We expect that XAI will be increasingly deployed in healthcare and careful design of XAI outputs which are clinically acceptable is important given the high stakes nature of medical decision making. T2 in clinical CMR is thought to be a sensitive marker for acute myocarditis and to our knowledge the heterogeneity of T2 distribution in this population has not been described. This imaging biomarker could aid the diagnosis of acute myocarditis and we postulate it may have prognostic implications for these patients.

## 5 Conclusions

We have evaluated AI/ML applications to CMR in the diagnosis of acute myocarditis. The studies described whilst classifying myocarditis images lack metadata and the majority differ in their aim from the clinical diagnostic objective which is to differentiate between distinct cardiac diseases that share similar clinical presentations. Whilst in some cases the image dataset is publicly available, often imaging datasets for ML in cardiovascular disease are not accessible for researchers and we anticipate that increasing availability of large cardiac imaging datasets will be leveraged to drive ML augmented diagnosis of cardiovascular diseases including myocarditis with a direct positive impact on patient care.
